# SuperSpot: coarse graining spatial transcriptomics data into metaspots

**DOI:** 10.1093/bioinformatics/btae734

**Published:** 2024-12-09

**Authors:** Matei Teleman, Aurélie A G Gabriel, Léonard Hérault, David Gfeller

**Affiliations:** Department of Oncology, Ludwig Institute for Cancer Research Lausanne, University of Lausanne, Lausanne 1011, Switzerland; Swiss Institute of Bioinformatics (SIB), Lausanne, Lausanne 1015, Switzerland; Agora Cancer Research Center, Lausanne 1011, Switzerland; Swiss Cancer Center Leman (SCCL), Switzerland; Department of Oncology, Ludwig Institute for Cancer Research Lausanne, University of Lausanne, Lausanne 1011, Switzerland; Swiss Institute of Bioinformatics (SIB), Lausanne, Lausanne 1015, Switzerland; Agora Cancer Research Center, Lausanne 1011, Switzerland; Swiss Cancer Center Leman (SCCL), Switzerland; Department of Oncology, Ludwig Institute for Cancer Research Lausanne, University of Lausanne, Lausanne 1011, Switzerland; Swiss Institute of Bioinformatics (SIB), Lausanne, Lausanne 1015, Switzerland; Agora Cancer Research Center, Lausanne 1011, Switzerland; Swiss Cancer Center Leman (SCCL), Switzerland; Department of Oncology, Ludwig Institute for Cancer Research Lausanne, University of Lausanne, Lausanne 1011, Switzerland; Swiss Institute of Bioinformatics (SIB), Lausanne, Lausanne 1015, Switzerland; Agora Cancer Research Center, Lausanne 1011, Switzerland; Swiss Cancer Center Leman (SCCL), Switzerland

## Abstract

**Summary:**

Spatial Transcriptomics is revolutionizing our ability to phenotypically characterize complex biological tissues and decipher cellular niches. With current technologies such as VisiumHD, thousands of genes can be detected across millions of spots (also called cells or bins depending on the technologies). Building upon the metacell concept, we present a workflow, called SuperSpot, to combine adjacent and transcriptomically similar spots into “metaspots”. The process involves representing spots as nodes in a graph with edges connecting spots in spatial proximity and edge weights representing transcriptomic similarity. Hierarchical clustering is used to aggregate spots into metaspots at a user-defined resolution. We demonstrate that metaspots reduce the size and sparsity of spatial transcriptomic data and facilitate the analysis of large datasets generated with the most recent technologies.

**Availability and implementation:**

SuperSpot is an R package available at https://github.com/GfellerLab/SuperSpot and archived on Zenodo (https://doi.org/10.5281/zenodo.14222088). The code to reproduce the figures is available at https://github.com/GfellerLab/SuperSpot/tree/main/figures (https://doi.org/10.5281/zenodo.14222088).

## 1 Introduction

Spatial transcriptomics enables researchers to simultaneously capture the spatial organization of cells within a tissue slice and their gene expression profile. Two main groups of spatial transcriptomic technologies have been developed. Sequencing-based technologies (e.g. 10x Visium, Visium HD) use spatially barcoded probes or oligonucleotide spots to capture mRNA molecules from a tissue section. Following sequencing, the spatial transcriptomic data can be mapped back to the original tissue section (Rodriques *et al.* 2019, Nagendran *et al.* 2023). Imaging-based technologies (e.g. Nanostring CosMx) rely mainly on fluorescence in situ hybridization or in situ sequencing to detect specific mRNA molecules within a tissue section, followed by high-resolution imaging and computational analysis to map the expression of hundreds to thousands of genes in their native tissue context (Chen *et al.* 2015, He *et al.* 2023, Shi *et al.* 2023). The captured mRNAs are assigned to spots after cell segmentation.

Historically, sequencing-based approaches were limited to a few thousand spots of roughly 10 cells, with some variability depending on tissue type, cell size, and section thickness (Du *et al.* 2023, Park *et al.* 2023). More recently, the resolution of these approaches has significantly increased allowing researchers to profile millions of spots/bins at almost single-cell resolution with the VisiumHD (Nagendran *et al.* 2023). Such large numbers of spots make it very challenging to run even standard analyses like identifying spatially variable genes. Imaging-based methods can reach sub-cellular resolution and profile hundreds of thousands of segmented cells (Chen *et al.* 2015). Akin to single-cell RNA-Seq (scRNA-Seq) both sequencing-based and imaging-based spatial transcriptomic data are very sparse (from 80% to 95% of zeros).

To simultaneously address size and sparsity issues in scRNA-seq data, metacells have been proposed, with tools like MetaCell, SuperCell, or SEACells (Baran *et al.* 2019, Ben-Kiki *et al.* 2022, Bilous *et al.* 2022, 2024, Persad *et al.* 2023). Metacells consist of disjoint groups of cells showing very high transcriptomic similarity which are aggregated together (Bilous *et al.* 2024). Metacell approaches reduce technical noise and computational resource requirements while preserving biological heterogeneity. The ratio between the number of cells and the number of metacells is defined as the graining level (*γ*) (Bilous *et al.* 2024). In many metacell workflows *γ* can be determined by the users, which is convenient for exploring different values of this parameter.

In this work, we expand the metacell concept to spatial transcriptomic data with the aim of simultaneously reducing the size and sparsity of such data by merging spots into metaspots and preserving both the spatial and transcriptomic information.

## 2 Results

**Figure 1.**
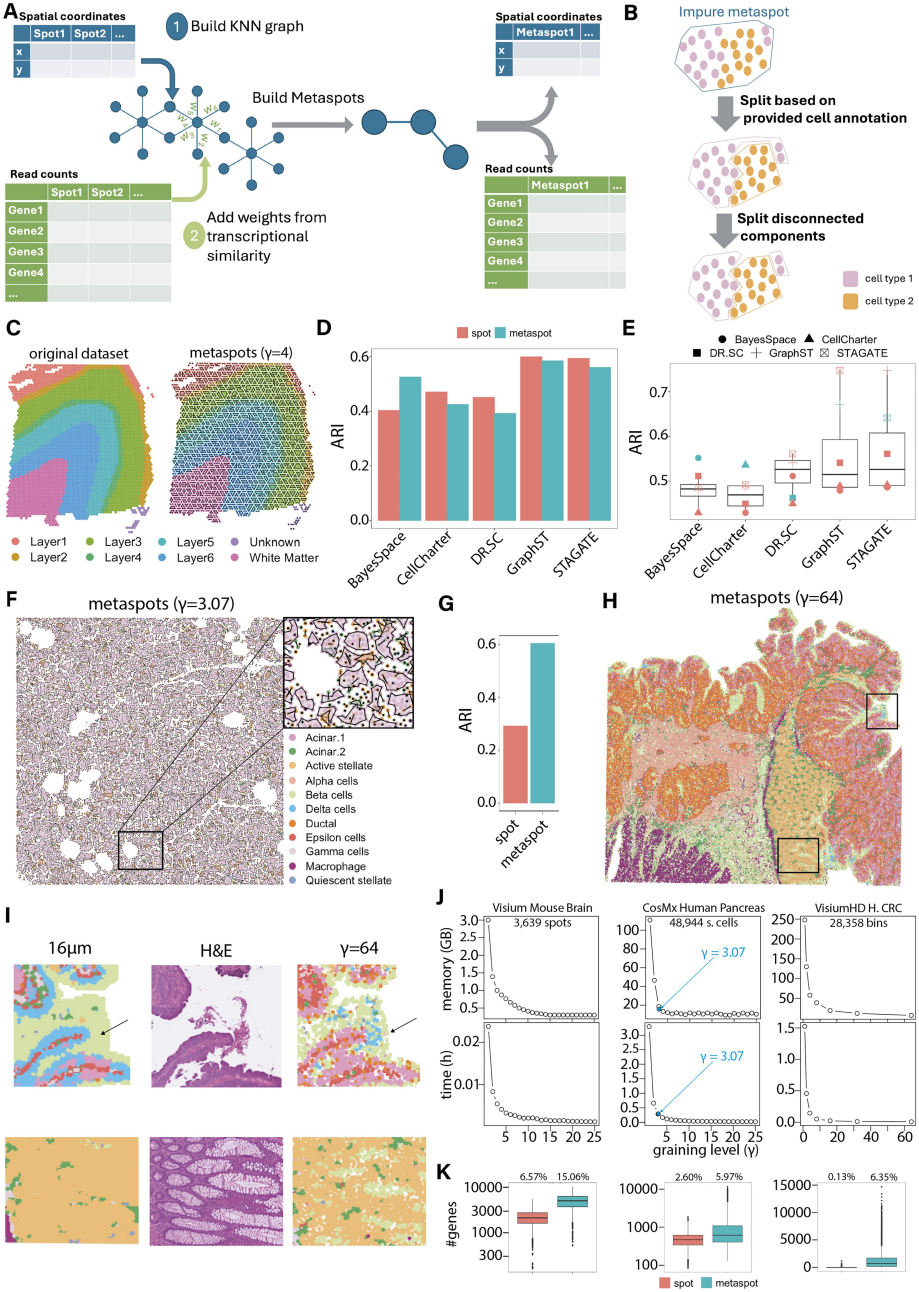
SuperSpot overview and applications. (A) Illustration of the SuperSpot pipeline to build metaspots in spatial transcriptomic data. (B) Splitting process of metaspots to guarantee both purity according to some annotation and spatial coherence. (C) 10x Visium mouse cortex dataset at the spot and metaspot (*γ* = 4) level. The spots and the metaspots are colored based on the layers of the cortex and white matter. The “Unknown” label corresponds to unannotated spots. (D) ARI scores of different spatial clustering methods with respect to the brain layer annotation at spot (red) and metaspot (blue) levels. The ARI score is computed as the mean over 10 runs with different seeds. (E) ARI scores between the clusters computed at the spot and metaspot levels by each clustering method (blue dots) and the clusters computed by different clustering methods at spot level (red dots). The ARI score is computed as the mean over 10 runs with different seeds. (F) Right slide of Nanos-tring CosMx human pancreas dataset at the metaspot (*γ* = 3.07) level. The spots and the metaspots are colored based on the cell types. (G) ARI scores of CellCharter with respect to the cell type annotation at spot (red) and metaspot (blue) levels on Nanostring CosMx human pancreas dataset. The ARI score is computed as the mean over 10 runs with different seeds. (H) VisiumHD Human Colorectal Cancer dataset. Colors show clusters obtained in transcriptomic analysis of metaspots and projected on the spatial coordinates of metaspots (*γ* = 64). Frames corresponds to the regions of interest for panel I. (I) Illustrations of clusters computed in 16 μm bins and in metaspots (*γ* = 64), compared to the H&E staining image. (J) Peak memory (GB) and elapsed time needed for computing normalization and spatially variable features in metaspots as a function of *γ* for the Visium Mouse Brain (3639 spots/33 538 genes, left), the Nanostring CosMx Human Pancreas (48 944 segmented cells/18 946 genes, center) and a 9·104x zoom of the VisiumHD Human Colorectal Cancer (H. CRC) (28 358 bins out of 8 731 400 2 μm bins/18 085 genes, right). Blue dots represent the final *γ* of 3.07 for the CosMx Human Pancreas after splitting impure metaspots. (K) Boxplot of the number of detected genes per spot (red) and metaspot (blue) for Visium Mouse Brain (*γ* = 4, left), Nanostring CosMx Human Pancreas (*γ* = 3.07, center), and VisiumHD Human Colorectal Cancer (*γ* = 64, right). Percentages above each boxplot correspond to the number of nonzero entries within the gene expressions matrix.

To adapt the concept of metacells to spatial transcriptomics, we developed the SuperSpot workflow. This workflow builds a K-Nearest Neighbor (KNN) graph on the coordinates of the spots and uses transcriptomic similarity as weight for the edges (Fig. 1A). For sequencing-based data generated with 10x technology we consider edges between adjacent spots (*K* = 6 for Visium, *K* = 8 for VisiumHD). For imaging-based data we start with *K* = 15. To avoid long-range connections between distant spots in sparse regions of the images, the top *F* (*F* = 40%) of connections based on Euclidian distances are removed from the graph. To compute the weights, Principal Component Analysis (PCA) is performed on the normalized count matrix. For each pair of connected spots in the graph, a transcriptomic distance is computed from the top 30 Principal Components space. The resulting distance is converted into a transcriptomic similarity measure by the function: *1—distance/max(distance)*. As in the SuperCell method (Bilous *et al.* 2022), the walktrap hierarchical clustering (Pons and Latapy 2005) is then applied on the graph to merge the spots into metaspots. Alternatively, for very large datasets, a faster and less memory intensive greedy algorithm is provided (Clauset *et al.* 2004). In this way, users can rapidly explore different *γ* without having to recompute the clustering for each *γ*. The metaspots are defined transcriptomically by aggregating raw counts and renormalizing them at the metaspot level, and spatially by computing the centroid of the spatial coordinates of the spots they consist of.

When a spot categorical annotation (e.g. cell types or niches) is provided, metaspots containing spots with distinct annotations can be split (Fig. 1B). To this end, metaspots are first split into smaller metaspots for each category of the annotation. To keep spatial coherence, we split a second time the resulting metaspots if they consist of disconnected components in the initial KNN graph. These two splitting steps induce a final *γ* smaller than the initial one.

To explore the impact of parameters *K*, *F*, and *γ*, we used the STARMAPplus Mouse Brain dataset (Shi *et al.* 2023). Supplementary Figure S1A and B show the distribution of distances in the KNN graph, and metaspots with and without filtering the top F long-range connections. Systematically, we observed that larger values for F reduce the maximum distance between cells within the same metaspot for most metaspots and increase the number of singletons (Supplementary Fig. S1C). Larger values for *K* result in a lower number of singletons but higher maximum distances (Supplementary Fig. S1D). Higher *γ* (i.e. lower number of metaspots) leads to larger maximum distances, as expected (Supplementary Fig. S1E).

To evaluate how metaspots can be used for downstream analyses, we applied different spatial clustering methods on a mouse cortex dataset generated with 10x Visium technology (Maynard *et al.* 2021), including GraphST (Long *et al.* 2023), STAGATE (Dong and Zhang 2022), CellCharter (Varrone *et al.* 2024), DR.SC (Liu *et al.* 2022), and BayesSpace (Zhao *et al.* 2021). This analysis was done both at the spot (3639) and metaspot (910) level with *γ* = 4 and without splitting the metaspots based on an annotation (Fig. 1C). The obtained clusters were compared to brain layer annotation of the spots using the Adjusted Rand Index (ARI—average over 10 rounds for each clustering algorithm with different random seeds). We observed similar results at the spot and metaspot resolution (Fig. 1D), indicating that metaspots can be used for clustering analyses. Additionally, the differences in the clusters computed at the spot and metaspot levels using the same algorithm were comparable to differences obtained when using different clustering algorithms at the spot resolution (Fig. 1E).

We then explored whether metaspots consist of spots with similar cell-type composition. To this end, we used a Human Breast Cancer tissue with heterogeneous cell type spatial distributions. Cell type proportion in each spot was predicted with Dampened Weighted Least Squares (DWLS) (Tsoucas *et al.* 2019) (Supplementary Fig. S2A), and the variability of the predicted cell-type proportions was computed for each metaspot. This variability was lower than for randomly aggregated spots (Supplementary Fig. S2B).

We next applied SuperSpot to a publicly available Nanostring CosMx dataset of a human pancreas (CosMx SMI Human Pancreas FFPE Dataset 2024) (48 944 segmented cells for 18 946 genes, Fig. 1F). We explored the metaspots obtained following the splitting procedure in Fig. 1B (final *γ* of 3.07 resulting in 15 943 metaspots) (Fig. 1F). In this dataset, the differences between cells and metaspots are mainly coming from the most abundant cell type (the Acinar.1 cell population, Supplementary Fig. S3A and B), resulting in several singletons and a broad distribution of metaspot sizes (Supplementary Fig. S3C). Expression values of differentially expressed genes obtained at the spot level between the two most prevalent cell types, Acinar.1 and Ductal, show higher and more homogeneous expression for the marker genes at the metaspot level (Supplementary Fig. S3D). We then compared the results of clustering of spots and metaspots with CellCharter which can be applied to both sequencing-based and imaging-based data. The ARI scores between clusters and cell type annotations were higher for metaspots built with the annotation-based splitting procedure of Fig. 1B than for spots (Fig. 1G).

We further explored whether metaspots are compatible with standard visualization at the transcriptomic level. To this end, we compared UMAP analysis performed on the spots and metaspots [after SCT normalization (Hafemeister and Satija 2019) followed by PCA dimension reduction]. We observed that cell types were equally distinguishable in both cases (Supplementary Fig. S3E), suggesting that metaspots can be used for visualization of spatial transcriptomic data at the gene expression level.

With the release of VisiumHD, metaspots provide an alternative to the grid-based pooling. We aggregated the 2 µm bins with *γ* = 64 on a Human Colorectal Cancer dataset. Considering the size of the data, we used fast greedy clustering to build the metaspots. When comparing the results of transcriptomic Louvain clustering performed on the default 16 µm bins with our metaspots, we noticed that clusters on metaspots better preserved some regions (Fig. 1H and I). We then investigated whether metaspots consisted of 2 µm bins from the same cell. From a mouse brain dataset with high enough resolution H&E staining slides, we observed cases where the metaspots could faithfully recapitulate single cells but also some differences (Supplementary Fig. S4A). We anticipate that integrating the H&E staining image in metaspot construction will increase the correspondence between metaspots and cells in such data (Polański *et al.* 2024).

We investigated how much metaspots impacted the computational resources needed to analyze spatial transcriptomic data. For different *γ*, we built metaspots with the procedure described in Fig. 1A. We then performed normalization and computation of spatially variable genes with the Seurat (Hao *et al.* 2024) R package. We observed a rapid decrease of both time and memory usage with higher *γ* for all three types of data (Fig. 1J). The number of detected genes per metaspot also increased at the metaspot level, demonstrating that metaspots reduce sparsity in spatial transcriptomic data (Fig. 1K).

Compared to metacells in scRNA-Seq data, the *γ* that can be reached in metaspots is lower when imposing the constraint of purity with respect to cell type annotation. This is especially the case for heterogeneous tissues containing a high diversity of cell types within the same regions where the graining level is by construction limited by the spatial distribution of the different cells. As such, we anticipate that metaspots will be especially useful for tissues containing relatively homogeneous regions, or technologies reaching sub-cellular resolution.

In summary, we extended the metacell concept to spatial transcriptomic datasets. Unlike approaches considering only spatial proximity to group spots/bins (Morabito *et al.* 2023), our metaspots show promise in maintaining spatial and phenotypic information and improve computational efficiency for downstream analyses of spatial transcriptomic data. Considering the size of the latest spatial transcriptomic data, even modest graining levels (e.g. *γ* ∼ 5) will be useful for reducing the computational burden associated with the analysis of such data.

## Supplementary Material

btae734_Supplementary_Data

## Data Availability

The STARMAPplus Mouse brain data is accessible from Zenodo (https://zenodo.org/records/8327576) as “well7_5”. The 10x Visium Mouse Cortex raw data is accessible from the Globus endpoint “jhpce#HumanPilot10x” (http://research.libd.org/globus) and the processed data from SpatialLIBD (Maynard *et al.* 2021) R package and website (https://research.libd.org/spatialLIBD/index.html). The 10x Visium Human Breast Cancer data is available from 10x’s website (https://www.10xgenomics.com/datasets/human-breast-cancer-block-a-section-1-1-standard-1-1-0). Nanostring CosMx Human Pancreas dataset and corresponding data are directly available from Nanostring’s website (https://nanostring.com/products/cosmx-spatial-molecular-imager/ffpe-dataset/cosmx-smi-human-pancreas-ffpe-dataset/). The 10x Visium HD data is available from 10x’s website: Human Colorectal Cancer sample (https://www.10xgenomics.com/datasets/visium-hd-cytassist-gene-expression-libraries-of-human-crc) and a mouse brain (https://www.10xgenomics.com/datasets/visium-hd-cytassist-gene-expression-libraries-of-mouse-brain-he).
