# Supplementary material for: SuperSpot: coarse graining spatial transcriptomics data into metaspots
Source: Bioinformatics. 2024 Dec 9;41(1):btae734. doi: 10.1093/bioinformatics/btae734 (PMC11725322; doi:10.1093/bioinformatics/btae734)
Supplement: btae734_Supplementary_Data [file btae734_supplementary_data.pdf]

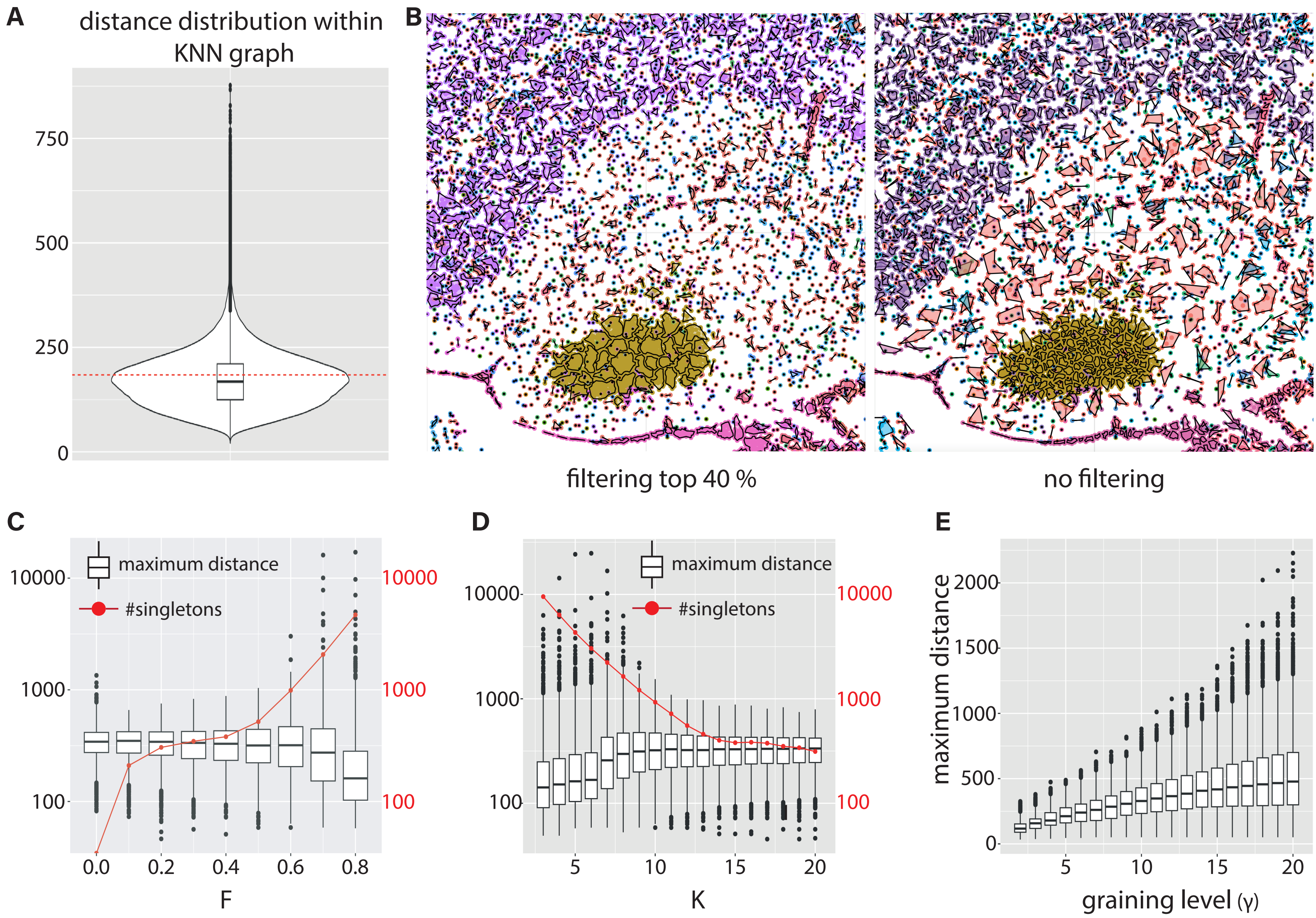

Supplementary Figure 1: Impact of parameters F, K and γ on SuperSpot.

A) Distribution of the edge distances in the KNN graph. The red dashed line represents the top F=40% connections that are removed by default. B) STARMAPplus Mouse Brain dataset where the colors represent the cell types. Metaspots are computed after removing the top F=40% long-range edges (left) and without removing any edge (right). C) Maximum distances within metaspots (boxplots) and number of singletons (connected red dots) as a function of F with γ=10 and K=16 (default). D) Maximum distances within metaspots (boxplots) and number of singletons (connected red dots) as a function of K with γ=10 and F=40% (default). E) Evolution of the maximum distances within metaspots as a function of the graining level γ.

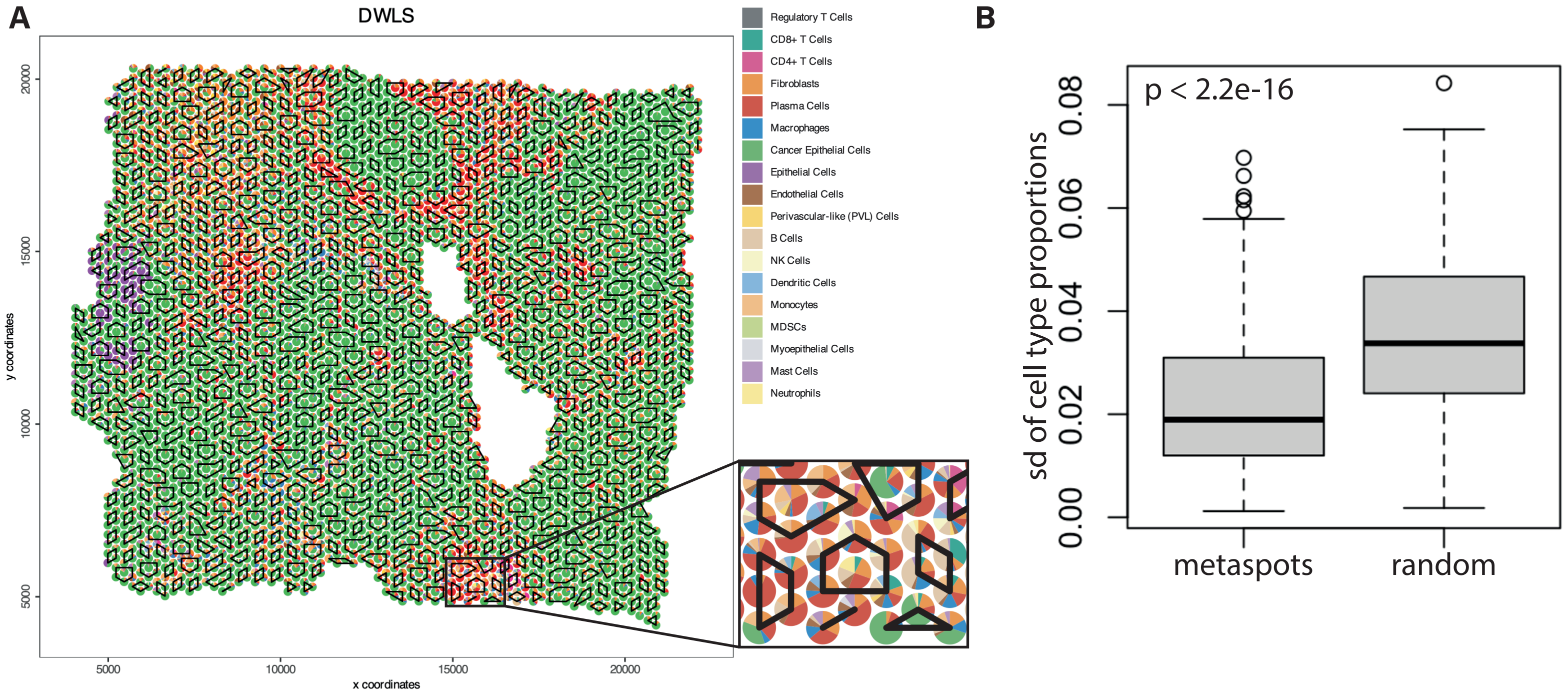

Supplementary Figure 2: Metaspots group similar spots in Visium data.
A) Predicted cell-type composition of spots by DWLS in a Human Breast Cancer dataset. B) Boxplots of the average standard deviation computed from the cell-type proportions of spots within the metaspots identified with SuperSpot, or within random metaspots. The P-value was computed with the T-test.

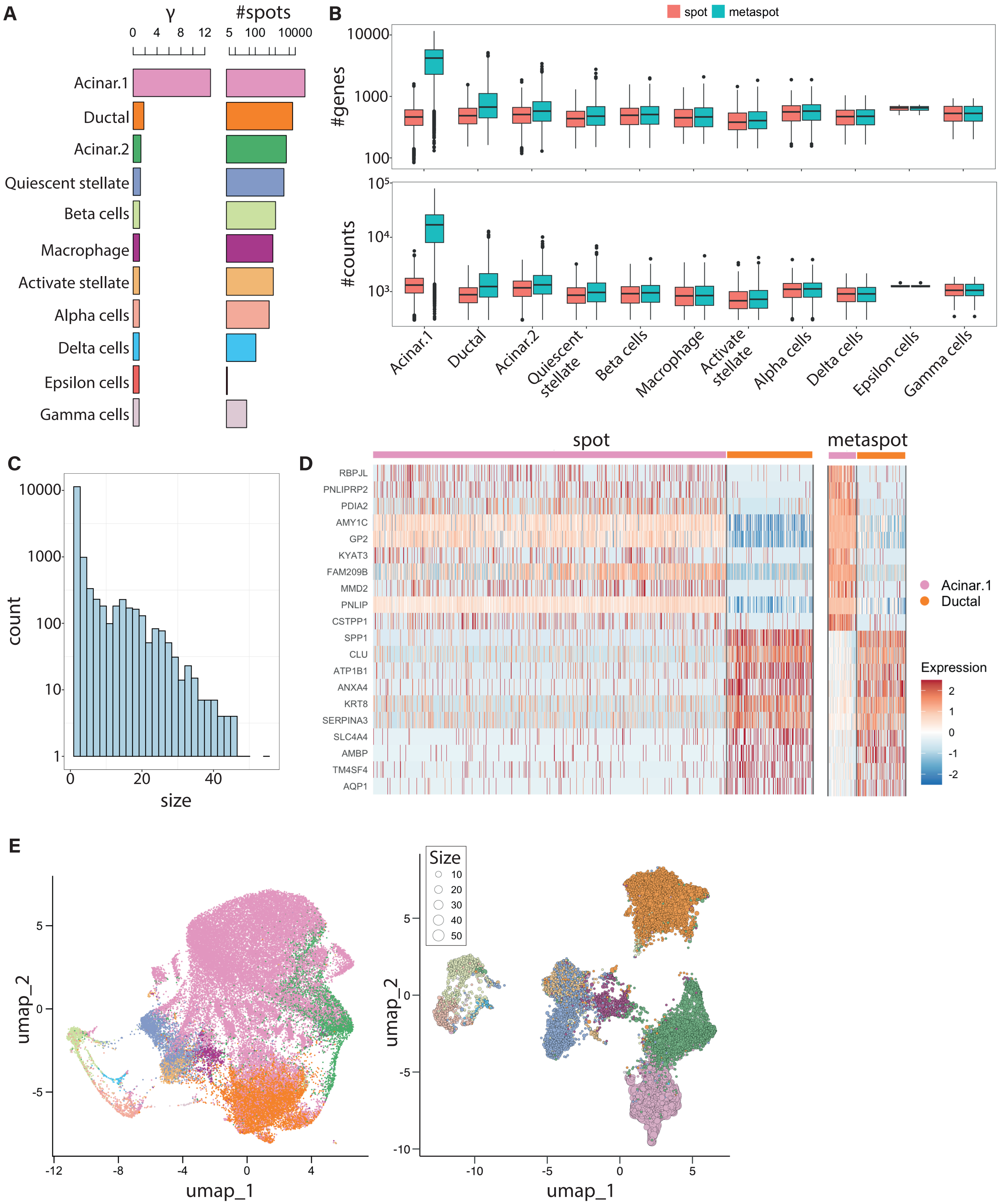

Supplementary Figure 3: Analysis of metaspots across different cell populations in the Nanostring CosMx human pancreas dataset.
A) Bar plots of the resulting γ and number of spots for each cell type. B) Boxplots of the number of genes and counts per spot and metaspot for each cell type. C) Histogram of the size of metaspots. D) Heatmap of the expression at the spot and metaspot levels of the top 10 differentially expressed genes identified at the spot level between Acinar.1 and Ductal. E) UMAP visualization of the original CosMx human pancreas dataset at the spot level (left) and at the metaspot level (right). For the UMAP at the metaspot level, the size of the dots is proportional to the number of spots contained in the metaspot. Colors correspond to the legend of panel A.

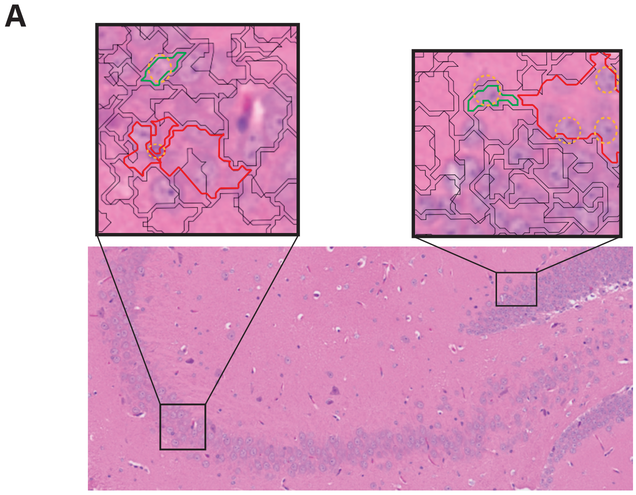

Supplementary Figure 4: Using SuperSpot to recreate cells from H&E staining.

A) Zoom in VisiumHD Mouse Brain H&E staining image with polygons indicating metaspots (γ = 64). The green boundaries show examples of metaspots matching cells from the H&E, and the red boundaries show examples of metaspots comprising multiple cells. Yellow dashed circles correspond to cells from the H&E staining.
